# Increasing the Effectiveness of Vaginal Microbicides: A Biophysical Framework to Rethink Behavioral Acceptability

**DOI:** 10.1371/journal.pone.0015501

**Published:** 2010-11-22

**Authors:** Stéphane Verguet, Bethany Young Holt, Andrew J. Szeri

**Affiliations:** 1 Institute for Health Metrics and Evaluation, University of Washington, Seattle, Washington, United States of America; 2 School of Public Health, University of California, Berkeley, and Coalition to Advance Multipurpose Innovations/Public Health Institute, Folsom, California, United States of America; 3 Department of Mechanical Engineering, University of California, Berkeley, California, United States of America; Aga Khan University, Pakistan

## Abstract

**Background:**

Microbicide candidates delivered via gel vehicles are intended to coat the vaginal epithelium after application. The coating process depends on intrinsic biophysical properties of the gel texture, which restricts the potential choices for an effective product: the gel first must be physically synthesizable, then acceptable to the user, and finally applied in a manner promoting timely adequate coating, so that the user adherence is optimized. We present a conceptual framework anchoring microbicide behavioral acceptability within the fulfillment of the product biophysical requirements.

**Methods:**

We conducted a semi-qualitative/quantitative study targeting women aged 18–55 in Northern California to assess user preferences for microbicide gel attributes. Attributes included: (i) the wait time between application and intercourse, (ii) the gel texture and (iii) the trade-off between wait time and gel texture. Wait times were assessed using a mathematical model determining coating rates depending upon the gel's physical attributes.

**Results:**

71 women participated. Results suggest that women would independently prefer a gel spreading rapidly, in 2 to 15 minutes (P<0.0001), as well as one that is thick or slippery (P<0.02). Clearly, thick gels do not spread rapidly; hence the motivation to study the trade-off. When asked the same question ‘constrained’ by the biophysical reality, women indicated no significant preference for a particular gel thickness (and therefore waiting time) (P>0.10) for use with a steady partner, a preference for a watery gel spreading rapidly rather than one having intermediate properties for use with a casual partner (P = 0.024).

**Conclusions:**

Biophysical constraints alter women's preferences regarding acceptable microbicide attributes. Product developers should offer a range of formulations in order to address all preferences. We designed a conceptual framework to rethink behavioral acceptability in terms of biophysical requirements that can help improve adherence in microbicide use ultimately enhancing microbicide effectiveness.

## Introduction

As women now account for 60% (75% between the ages of 15 and 24 [1]) of the 22.4 million infected subjects in sub-Saharan Africa [1], a new approach such as a vaginal microbicide empowering women against HIV infection has become a necessity. Microbicides are chemical agents used intravaginally with a goal to protect users against sexually transmitted infections (STIs) including HIV [2]. Multipurpose prevention microbicides, also referred to as ‘combinations’, are in development which may provide protection from HIV, some sexually transmitted infections, as well as unplanned pregnancy and other reproductive tract infections [3]. They can be classified according to different mechanisms of action: non-specific microbicides, moderately specific microbicides, highly specific anti-HIV agents, etc [4]. After disappointing results for all the efficacy trials conducted evaluating detergent and polyanionic formulations [5]–[7], the CAPRISA 004 trial, evaluating an antiretroviral drug-based microbicide [8], recently proved to be successful with a 28–54% range of effectiveness depending on the user's adherence [9]. About 77 other microbicide candidates are currently in the clinical or preclinical pipeline [10]. Multiple challenges lie ahead in terms of clinical trials and product development [11], [12] among which the issue of the user's adherence [9], [13].

Recently, research has paid great attention to the delivery systems for the active ingredients such as gels or intravaginal rings, and has focused on aspects of coital dependency, compliance, cost and adaptability to large-scale production for instance [14]. Indeed, the delivery systems are crucial: in order for the active ingredients to work effectively, they must be adequately distributed to target tissue sites where disease transmission or pathogenesis occurs in the vagina [15]. In the particular case of gel vehicles, the effectiveness of the microbicide would depend on fundamental biophysical properties of the gel texture, such as the coating process [15] as well as on gravity, adhesion, and shearing forces from surrounding tissues [16]. At the same time, a microbicide gel will not be effective unless applied consistently and correctly by the user: this is especially important in clinical trials [17] where a fall-out in adherence can significantly ‘dilute’ efficacy measurements [13], [18] as observed in the CAPRISA 004 trial [9]. In that regard, biophysical properties and the social/behavioral attributes of microbicide products are thus intrinsically linked. For example, how quickly the gel spreads, or its viscosity, will in turn have potential impact on the adherence by the intended consumer: a product that is highly viscous may require several hours to spread effectively, thus impacting the waiting time before protected sexual intercourse [19]. Longer waiting times may be less well adhered to, or better, depending on the context and the user. Conversely, a less viscous product could spread more rapidly and require a shorter waiting period [19], but would likely be more messy. The preference over this range of constrained possibilities may differ by user or by specific situation.

Most studies have assessed acceptability using either candidate microbicides [20]–[23] or surrogate products [24], [25]. In addition, because microbicides are an evolving technology, earlier studies focused on soliciting input from individuals about hypothetical products using traditional epidemiological and behavioral research methodologies [26]–[28]. Some studies have focused on some specific population segments such as women at high risk [29], adolescents [30] etc.; on the context of sexual relationships [31]; or on the involvement of male partners [32], [33]. Others have looked at characteristics of the product itself and precisely on the ‘vehicle-associated factors’ such as the product's ‘color’, ‘smell’, consistency’, ‘leakage’ potential [34], [35]. Previous research has applied mixed methods using both quantitative and qualitative data [34], [36] or a more market-oriented research method with ‘conjoint analysis’ [36], [37] to study hypothetical users' preferences. Reviews of microbicide acceptability research have emphasized the social-cultural factors [38] and recommended the use of a comprehensive and integrated approach assessing ‘vehicle-associated’, ‘application-associated’ and ‘use-associated’ factors [39]. However, very little work to this day [40], [41] has started to explore in detail what biophysical properties of microbicide gels women are likely to prefer, given the range of *realistic* possibilities. In other words, how do constraints imposed by biophysical requirements alter the preferences users might have about microbicides?

We introduce through this study a new conceptual framework to assessing microbicide acceptability and use by evaluating the interplay between social and biophysical acceptability. Specifically, we integrate a well-established semi qualitative-quantitative framework [34], [36] with the biophysical reality of the gels described by a mathematical model of vaginal wall coating [19]. Essentially, our goal is to assess the trade-offs potential users would make between the thickness (viscosity) of the gel and the wait time needed for the gel to coat the vaginal epithelium (a proxy for the waiting time before sexual intercourse). The viscosity of the gel both provides the texture of the gel and governs the kinetics of the flow. It provides the physical attribute that links user preferences with physical functionality. We first ask in a series of questions about desirable attributes (thickness and coating time) independently. Next we explore changes in the preferences that emerge when biophysical constraints, assessed with the computer model [19], are taken into account. *The concept of a ‘constrained’ approach to acceptability is the novelty of this research*. Our methodology could be applied to the assessment of a variety of microbicide gel formulation attributes, for those targeting HIV only or those with multiple prevention targets, such as unintended pregnancy, HIV, and other infections. It could also further be extended to the field of biomedical interventions for HIV prevention and reproductive health.

## Materials and Methods

### Ethics statement

The University of California Berkeley Committee for the Protection of Human Subjects specifically approved the study. All participants read and signed a consent form approved by the University of California Berkeley Committee for the Protection of Human Subjects prior to participation. Participants received a small stipend.

### Methods

Both qualitative and quantitative data were collected to assess user acceptability. Eligible subjects for this study were women aged 18–55 years old, who had had heterosexual sexual intercourse in the previous 12 months. To assure a heterogeneous sample, participants were recruited from various venues in Northern California, including junior colleges that serve low-income, inner city communities, universities and colleges with middle- and upper-income student populations, community organizations and clinics serving immigrant and low-income populations. Various recruitment methods were used, including fliers posted throughout college campuses, in clinics, and other community venues, and announcements in classes, campus newspapers, and list-serves.

Focus group discussions (N = 71) were administered by a trained interviewer and audio-recorded and then transcribed. The discussions lasted approximately 60 minutes and were conducted in English. Transcripts were analyzed and coded to ascertain common themes using the qualitative data analysis software program ATLAS.ti (Version XX, The Knowledge Workbench. Berlin, Scientific Software Development Inc.). Because of the responsive nature of the discussions, each focus group was unique, thus the actual coding list was refined as the coding took place. Upon completion of the coding, a second researcher examined both the final coding list and the actual coding of each transcript. Any coding discrepancies were discussed between the two researchers and coding was modified according to their agreement. The discussions focused on women's attitudes towards existing sexual and reproductive health products, including HIV/STI and pregnancy prevention methods and lubricants, attitudes towards using a new STI prevention product, self-reported risk factors for HIV, STIs, and pregnancy. We also explored inferred microbicide preferences and how women would make trade-offs in terms of their preference among products with different biophysical characteristics. Participants were presented with a 5-minute description of microbicides, and information of how microbicides might be formulated and used. To help women visualize the distinction between a ‘highly viscous’ gel and a ‘less viscous’ gel, the interviewer showed participants several different over the counter (OTC) products currently available and applied the products to her own hand to illustrate viscosity. To illustrate a highly viscous gel, Vagisil Regular Strength Anti-Itch (©Combe, Inc.) cream was demonstrated and to illustrate a less viscous gel, KY Jelly (©Johnson & Johnson, Inc.) Personal lubricant was demonstrated. Next, the women completed a brief questionnaire. The questionnaire had three main components. The first component assessed self-reported HIV/STI risk status and use of preventive methods. The second section explored microbicide preferences and how women would make trade offs in terms of their preferences among products with different biophysical characteristics. The third survey component recorded socio-demographic variables. The survey was administered by a trained interviewer and was self-administered following the focus group discussions.

This present work focuses on the second component of the questionnaire, in which we asked women three sets of questions to explore the relationship between product acceptability and biophysical properties of potential microbicide products. The questions of this section were driven by the mathematical model [19] used to assess the biophysical characteristics of the product. The model was used to determine how coating rates would change when physical attributes of the gel were changed. First, we asked a ‘time’ question to assess how likely (on a scale ranked from 1 to 7) women would use each of three microbicides which varied by how much time before sexual intercourse they needed to be applied. The three distinct times, suggested by the model and physical attributes in the ranges of candidate microbicides, were: 1/ 2–15 minutes; 2/ 1 hour; 3/ 10 hours. Second, we asked a ‘thickness’ question, about how likely (on a scale ranked from 1 to 7) would women be, to use each of three microbicides varying by their feeling of thickness upon application. The three distinct feelings were: 1/ watery; 2/ slippery; 3/ thick. Third, we asked a ‘constrained’ question, about how likely (on a scale ranked from 1 to 7) women would use each of three microbicides. The latter microbicides were three distinct combinations of ‘time’ and ‘thickness’: 1/ a watery microbicide for which the user would have to wait for 2–15 minutes before sexual intercourse; 2/ a slippery microbicide for which the user would have to wait 1 hour before sexual intercourse; 3/ a thick microbicide for which the user would have to wait 10 hours before sexual intercourse.

The time of spread of the gels was translated from viscosity data from gels such as KY Jelly (©Johnson & Johnson, Inc.), Carraguard (©The Population Council, Inc.), and HEC (placebo gel used in the CAPRISA 004 trial [9]) etc. obtained from David Katz and coworkers at Duke University (David Katz, Private Communication) through a timescale proportional to *η*, where *η* is a representative viscosity of the gel. The thickness of the gels was expressed into sensations of ‘watery’, ‘slippery’ and ‘thick’ from physical experiences with commercial gels and was linked to three different viscosity scales coming from the common physical sense that a watery gel has a low viscosity, that a thick gel has a high viscosity etc. Lastly, the ‘constrained’ series of questions was simply revealing the links among attributes of gels that derive from biophysical constraints (i.e. if a gel is thick, it is going to have a high viscosity and therefore it is going to take a longer time to spread onto the vaginal epithelium).

Analysis of the questionnaire data was accomplished using R statistical package R 2.10.1 (The R Project for Statistical Computing, 2009. http://www.r-project.org/).

## Results

### Demographics and behavioral characteristics of the study population

Demographic and behavioral characteristics of the study population are reported in table 1. The majority of the women who participated were White (36%) followed by ‘mixed race’ (23%) and Hispanic (20%). Slightly less than half of the sample was under 30 years of age (46%) and about a third of the women reported having had casual sex partners in the past year (31%). About half (51%) of the women reported having ever had an unplanned pregnancy and 31% reported ever having had an STI. While 93% reported ever using condoms and 79% reported ever using condoms in combination with a hormonal method for birth control, focus group discussions with these women suggested that condom use was inconsistent, especially with longer-term partners.

**Table 1:** Demographic and behavioral characteristics of the study population (N = 71).

Characteristic	Number of subjects*	(%)
**Age**		
>30 years old	38	54
≤30 years old	32	46
**Race/Ethnicity**		
African American	8	12
Asian	3	5
Hispanic	14	20
Native American	2	3
White	24	36
Mixed	15	23
**Employment Status**		
Full time	24	38
Part time	16	25
Unemployed	21	33
**Relationship Status**		
Married	21	32
Regular boyfriend/partner	24	36
Casual partner	21	31
**Prevention methods used for STI or pregnancy prevention**		
Male condoms	66	93
Female condoms	5	7
Hormonal contraception	60	85
Hormonal contraception plus condoms	56	79
Other (e.g., spermicide, natural, etc)	39	55
**History of STI diagnosis**	22	31
**History of an unplanned pregnancy**	36	51

*Note: data are missing for up to 7 women because of failure of some women to complete specific questions in the questionnaire.

### Quantitative analysis

**Figure 1:**
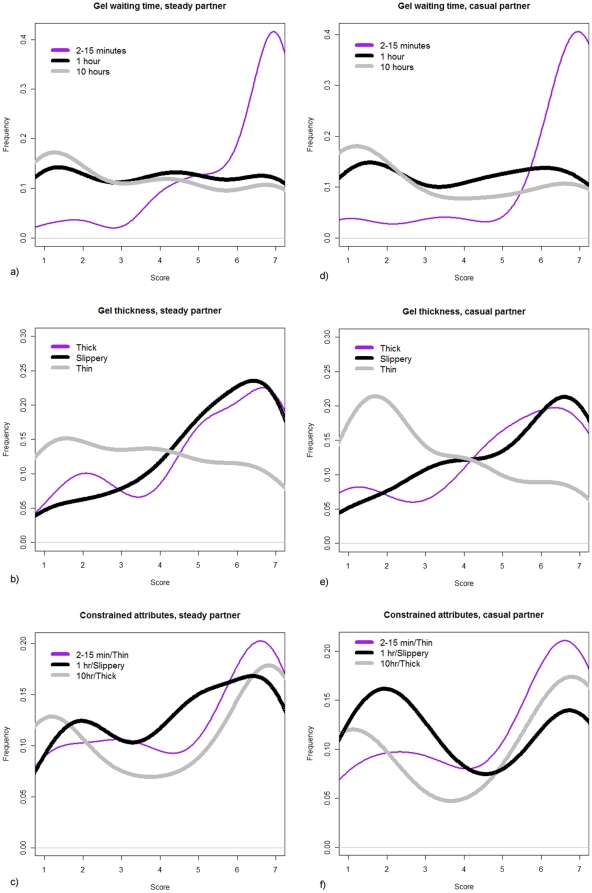
Distributions for the scores to each of the biophysical attribute questions. The distributions are for both steady and casual partners. a/ ‘Time steady’; b/ ‘Thickness steady’; c/ ‘Constraint steady’; d/ ‘Time casual’; e/ ‘Thickness casual’; f/ ‘Constraint casual’.

**Figure 2:**
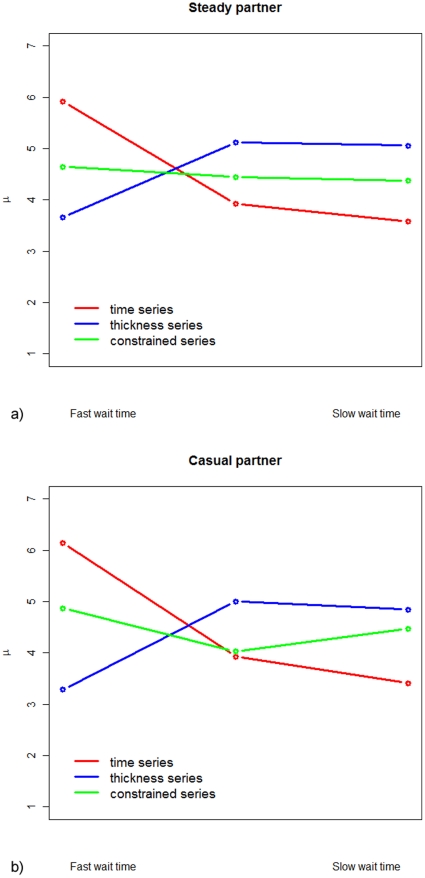
Mean scores for each of the biophysical attributes questions. The mean scores are for both steady and casual partner. a/ Steady partner; b/ casual partner.

The distributions of the answers (scores) corresponding to the series of questions ‘time’, ‘thickness’ and ‘constrained’ for use with a steady partner and a casual partner are presented on figure 1. Figure 2 displays the population means for the latter answers. The answers were further analyzed using paired *t*-tests for the means. We compared the scores of paired answers within each series of questions for steady vs. casual partner. For each subject *j*, each score was noted *X_i,j_* (*i* = *1*, *2*, *3* [1 = 2–15 min, 2 = 1 hr, 3 = 10 hr for the ‘time’ series]; *j* = *1*,*…*, *71*), leading to three answers *X_1,j_*, *X_2,j_* and *X_3,j_*. We then estimated the 3 differences *a*, *b* and *c* for each pair of scores: *D_a,j_* = *X_1,j_−X_2,j_*, *D_b,j_* = *X_1,j_−X_3,j_* and *D_c,j_* = *X_2,j_−X_3,j_* and calculated their population means: 
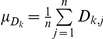
, where *k* = *a*, *b*, or *c* and *n* is the number of answers. The *t*-test statistic was then given by 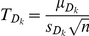, with the standard deviation 
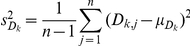
. The corresponding 95% confidence intervals and *p*-values were then derived. Table 2 collects the results.

**Table 2:** Results of the paired *t*-tests.

Paired answers	Number of answers*	Mean difference *μ_D_* (95% CI)	*p*-value
**Steady Partner**			
*Time series*			
***μ_Da_ = μ_X{2–15 min}_−μ_X{1 hr}_***	67	2.03 (1.48–2.58)	<0.0001
***μ_Db_ = μ_X{2–15 min}_−μ_X{10 hr}_***	66	2.38 (1.76–3.00)	<0.0001
***μ_Dc_ = μ_X{1 hr}_−μ_X{10 hr}_***	67	0.31 (−0.28–0.91)	0.295
*Thickness series*			
***μ_Da_ = μ_X{watery}_−μ_X{slippery}_***	36	−1.69 (−2.59–−0.80)	<0.0001
***μ_Db_ = μ_X{watery}_−μ_X{thick}_***	37	−1.67 (−2.43–−0.93)	<0.0001
***μ_Dc_ = μ_X{slippery}_−μ_X{thick}_***	66	−0.05 (−0.64–0.55)	0.879
*Constrained series*			
***μ_Da_ = μ_X{2/15 min/watery}_−μ_X{1 hr/slippery}_***	66	0.23 (−0.35–0.80)	0.434
***μ_Db_ = μ_X{2/15 min/watery}_−μ_X{10 hr/thick}_***	65	0.26 (−0.51–1.03)	0.501
***μ_Dc_ = μ_X{1 hr/slippery}_−μ_X{10 hr/thick}_***	65	0.03 (−0.59–0.65)	0.921
**Casual Partner**			
*Time series*			
***μ_Da_ = μ_X{2–15 min}_−μ_X{1 hr}_***	41	2.24 (1.39–3.09)	<0.0001
***μ_Db_ = μ_X{2–15 min}_−μ_X{10 hr}_***	38	3.03 (2.15–3.90)	<0.0001
***μ_Dc_ = μ_X{1 hr}_−μ_X{10 hr}_***	39	0.49 (−0.32–1.29)	0.227
*Thickness series*			
***μ_Da_ = μ_X{watery}_−μ_X{slippery}_***	36	−1.22 (−2.17–0.28)	0.016
***μ_Db_ = μ_X{watery}_−μ_X{thick}_***	37	−1.32 (−2.15–−0.50)	0.002
***μ_Dc_ = μ_X{slippery}_−μ_X{thick}_***	38	−0.13 (−0.81–0.54)	0.695
*Constrained series*			
***μ_Da_ = μ_X{2/15 min/watery}_−μ_X{1 hr/slippery}_***	37	1.00 (0.14–1.86)	0.024
***μ_Db_ = μ_X{2/15 min/watery}_−μ_X{10 hr/thick}_***	35	0.49 (−0.60–1.57)	0.908
***μ_Dc_ = μ_X{1 hr/slippery}_−μ_X{10 hr/thick}_***	35	−0.66 (−1.66–0.35)	0.192

The paired *t*-tests are for the mean difference between two answers for each of the three series of questions i.e. ‘time’, ‘thickness’ and ‘constrained’ series, for both a steady and casual partner.

*Note: not all women reported having a casual partner and data are missing for up to 6 women because of failure of some women to complete specific questions in the questionnaire.

Results indicate that users, whether with a steady or casual partner, would prefer a gel that spreads very fast of the order of 2 to 15 minutes as compared with 1 hour or 10 hours (p<0.0001). Likewise, they prefer a gel that is thick or slippery, as compared to watery (p<0.02). Figure 2 and table 2 illustrate the preferences when the subjects were asked the questions independently (i.e. without making a trade-off). Subsequently, if asked the question ‘constrained’ by physical reality, the subjects in a steady relationship tended to drop the unconstrained preferences for a thick gel and for a gel that spreads rapidly. On average, the same subjects present a more uniform distribution of preferences for any of the synthesizable gels (p>0.10) (see figure 2 and table 2). On the contrary, the data indicate that subjects in casual relationship would choose a gel that spreads rapidly though watery rather than a gel that has intermediate properties (p = 0.024), without discarding a thick and long spreading gel (p = 0.908) (see figure 2 and table 2).

### Qualitative analysis

Consistent with the quantitative findings, the qualitative data indicates that regardless of relationship status, women would prefer a product they can use spontaneously (i.e., within 2–15 minutes after application) and one that is highly viscous, or thick and not messy. However, when asked to make a trade-off between a product they could use shortly after application but was less viscous, similar to KY Jelly, compared to a thicker, more viscous product with less leakage that would require application several hours ahead of use to be effective, we saw differences according to relationship status consistent with the quantitative data. The findings suggest that among women in casual relationships, if asked to make a trade-off between viscosity and wait time, the priority for such women overall is to have a product which could be effective quickly (little wait time), regardless of viscosity. Indeed, when probed further, many women reported that a less viscous microbicide could even be appealing if it could enhance pleasure, such as a ‘warming’ gel or lubricant. Women in steady relationships were more concerned about the effectiveness of the product for prevention of pregnancy or STIs/HIV as it would be easier for them to plan ahead compared to women being in a casual relationship.

## Discussion

We designed a conceptual study where for the first time the behavioral acceptability of microbicide gels is realistically constrained and interpreted through the biophysical reality of the gels themselves. Specifically, we conclude that at the population level there is a fairly uniform spectrum of preferences for gels of different thicknesses (and so wait times) for women in a steady relationship, and a preference for a gel that spreads very fast as compared with a gel having intermediate properties for women in a casual relationship. In that sense, developers should offer a range of formulations in order to address the preferences of all users and therefore increase adherence. Though our results are not exhaustive due to the limitations of the sample size (N = 71), and although we would do well to incorporate a number of other attributes, the work here still introduces a new approach in the field. One could now use the latter approach to broaden the conversation and include questions with more characteristics that can govern gel coating such as pH, temperature [42] or interference with sexual intercourse [43] etc. Also, one should carry out such a new approach with different target populations from racially and socio-economically diverse communities and geographical regions, and most particularly with women at high risk for HIV/STIs.

The approach is novel as it presents for the first time a critical biophysical framework in which to rethink the acceptability of microbicide gel vehicles. The latter framework would ensure that end users like the gels' features offered by market developers, identifying individualized prevention strategies and generating the highest usage rates. It will help refine and tailor the microbicide gels' application instructions given to participants of clinical trials, and will help design future products that can achieve greater compliance rates. This is important, as poor adherence can contribute to the lack of effectiveness or reduced effectiveness observed in the clinical trials [9], [13], [18]. In particular, one could use this framework to study the trade-off between the user's preferences for microbicide physical attributes and adherence, in looking at the sensitivity of adherence patterns to biophysical attributes, revealing who uses microbicides correctly and consistently, and which factors enhance or constraint such use. Ultimately, such a better understanding of users' perspectives on the biophysical and behavioral acceptability of the product can contribute to more realistic designs for the future.

This work fits well into the new era of conceptualizing a mechanistic model to guide microbicide development [44] and with recent biophysical work on linking gel deployment and distribution in the human vagina to the user's acceptability [43]. In the meantime, we hope to duplicate and adapt this idea of constraints secondary to biophysical considerations for thinking about the acceptability of other drug delivery systems in the field of sexual and reproductive health. For instance, a first step in the field of microbicides would be to look at intravaginal rings that can stay in place for periods up to three months and are likely to show greater adherence [14]. One could envision asking similar unconstrained and constrained preference questions, where this time the discussion focused on the specific delivery method for the microbicide. Likewise, this framework could be duplicated within recent acceptability work of rectal microbicides [45].
